# Long-term experiences with high-energy shock wave therapy in the management chronic phase Peyronie’s disease using two different electromagnetic lithotripters

**DOI:** 10.1007/s00345-024-04792-x

**Published:** 2024-03-07

**Authors:** Jens J. Rassweiler, W. Scheitlin, A. S. Goezen, F. Radecke

**Affiliations:** 1 Chair of Urology and Andrology, Danube Private University, Steiner Landstraße 124, 3500 Krems-Stein, Austria; 2 Department of Urology, SLK Kliniken Heilbronn, Heilbronn, Germany; 3 Department of Urology Medius-Kliniken Ruit, University of Tübingen, Ostfildern-Ruit, Germany

**Keywords:** Extracorporeal shock wave therapy, Peyronie’s disease, acute and chronic phase, Induratio penis plastica, Low-intensity ESWT, High-intensity ESWT, Non-surgical treatment options

## Abstract

**Background:**

Extracorporeal shock wave lithotripsy represents one option for the non-surgical management of Peyronie’s disease. Despite promising results, several questions are still pending. We want to present the long-term results of a retrospective study using high-energy extracorporeal shock wave lithotripsy.

**Material and methods:**

We evaluated retrospectively 110 patients treated between 1996 and 2020 at the Department of Urology, SLK Kliniken Heilbronn for chronic phase Peyronie’s disease using two electromagnetic lithotripters (Siemens Lithostar Plus Overhead Module, Siemens Lithoskop) applying high-energy shock waves under local anesthesia and sonographic or fluoroscopic control. A standardized questionnaire focused on the change in pain, curvature, sexual function and the need of penile surgery.

**Results:**

In 85 of the 110 patients (mean age 54 years) we had sufficient data for evaluation. The median follow-up was 228 (6–288) months. There were no significant complications. Pain reduction was achieved in all patients, 65 (76%) patients were free of pain. Improvement of penile curvature was achieved in 43 patients (51%) ranging from 25% improvement (deflected angle < 30°) to 95% (angle 30–60°). 59 patients (69%) reported problems with sexual intercourse, 40 of those (68%) reported improvement. Only 9 (10.5%) patients underwent surgical correction. We did not observe any significant differences between both electromagnetic devices with stable long-term results.

**Conclusions:**

High-energy shock wave therapy delivered by two standard electromagnetic lithotripters is safe and efficient providing stable long-term results. In cases with significant plaque formation, the concept of high-energy ESWT should be considered in future studies.

## Introduction

Peyronie’s disease (PD) is an acquired connective tissue disorder characterized by fibrosis of the tunica albuginea resulting in the development of penile deformity, penile pain and penile shortening [1]. The prevalence of PD ranges between 3 and 20% with a higher incidence in diabetic men [2]. The pathophysiology of PD still remains unclear. However, it is believed that microvascular trauma, initiated by penile damage during sexual activity or due to repetitive minor trauma, leads to increased proliferation of fibroblasts and recruitment of profibrotic mediators with an excessive deposition of collagen. This remodels into a dense fibrotic plaque causing the onset of penile curvature [1–3].

Two phases of the disease can be distinguished. The first is the active inflammatory phase (acute phase), which may be associated with painful erections and a palpable nodule or plaque in the tunica of the penis. Typically, but not invariably, a penile curvature begins to develop. The second is the fibrotic phase (chronic phase) with the formation of hard, palpable plaques that can calcify, with stabilization of the disease and of penile deformity [1–3]. Penile plaque formation can result in a curvature, which may impair sexual intercourse.

Therapeutic options include oral medication, intralesional therapy, topical treatment, and surgery. None of the tested oral substances was able to change the course of disease, however, phosphodiesterase-5-inhibitors were able to reduce pain [1–3]. The best results have been achieved by intralesional injection of Collagenase of Clostridium histolyticum. However, the substance has been withdrawn from the European market. Moreover, rupture of the tunica albuginea has been reported [4].

Extracorporeal shock wave therapy (ESWT) for chronic phase Peyronie’s disease was introduced at the end of the last century [5, 6]. This was followed by a positive randomized trial [7]. However, other RCT studies were only able to demonstrate pain reduction in patients treated by ESWT [8, 9].

Whereas early studies used classical lithotripters (i.e. Siemens Lithostar Overhead Module, Lithostar multiline, EDAP LT02, Wolf Piezolith 2500, Dornier Compact Delta III) applying higher levels of shock wave energy [5, 6, 10–14], in the RCTs a low-energy setting applied by devices mainly developed for orthopedic indications (i.e. Storz Duolith SD, Wolf Piezoson) was used [7, 9]. Further, non-randomized studies used low-energy devices as well, such as Wolf Piezoson, Storz Minilith, Storz Duolith SD1, Dornier Epos, Omnispec ED1000 [15–18].

The aim of low-intensity ESWT is not to break the plaque but to activate a remodeling of the plaque itself by micro-damages to the tunica albuginea and to the plaque. Furthermore, it has been demonstrated that ESWT is useful in the acute phase, for pain management also in the long-term [21]. Accordingly, the current EAU guidelines only recommend Li-ESWT for the management of pain [2].

However, there is still the open question, whether significant, even calcified plaques could be sufficiently treated high-intensity ESWT using standard lithotripters. Recently, also small devices applying higher intensity levels have been introduced by some manufacturers (ie. Storz Medical Duolith SD1-ultra). We want to present our long-term results treating more than 100 patients during the last 20 years with high-energy ESWT using two different electromagnetic lithotripters and compare it with the existing long-term studies [19–21].

## Material and methods

### Patients (Table 1)

**Table 1:** Characteristics of patients suffering from Peyronie’s disease treated by high-energy shockwave therapy using two different electromagnetic lithotripters

Parameter	Overall	Lithostar overhead module (group 1)	Lithoskop (group 2)
Patients treated	110	64	46
Age (years)	54	53	55
Hypertension	15 (14%)	9	6
Diabetes mellitus	21 (19%)	12	9
Duration of disease (months)	14 (10–24)	15	13
Pre-treatment with potaba	81	59	20
With tocopherol (Vit. E)	12	8	4
With taldalafil	1	–	1
With cortisone-injection	4	4	–
With radiotherapy	4	4	–
With ESWT	4	2	2
Patients lost of follow-up	13	9	4
Patients excluded due to pretreatment (ESWT, radiotherapy)	8	6	2
Patient excluded due to < 3 sessions	4	3	1
Patients evaluated	85	46	39
Median time of follow-up (months)	228 (6–288)	251 (288–226)	114 (6–226)

^a^Apart from the median time of follow-up no significant statistical differences between the two cohorts

From July 1996 to June 2020, we treated at the Department of Urology SLK Kliniken Heilbronn 110 men suffering from chronic phase Peyronie’s disease using two electromagnetic lithotripters: from July 1996 to December 2001, we used the Overhead Module of the Lithostar Plus (Siemens, Erlangen, Germany) in 64 patients (Group 1). From January 2002 to June 2020, we used the Siemens Lithoskop (Siemens, Erlangen, Germany) in 46 patients (Group 2). All patients have been registered immediately after the first treatment in our data base (Microsoft Excel), but the results have been acquired retrospectively.

81 of the 110 patients were unsuccessfully pretreated with Potassium Para-aminobenzoate (POTABA-Glenwood, Cheplapharm, Greifswald, Germany). 12 patients took tocopherol (Vitamine E) without significant improvement. One patient was under taldalafil. 4 Patients had undergone local radiotherapy, and three had previous ESWT. Median duration of the disease was 14 (10–24) months. 15 (14%) of patients suffered from hypertension and 21 (19%) from diabetes mellitus. The median age was 54 (27–71) years. There were no statistical differences between the two groups (Overhead Module vs. Lithoskop).

25 patients were excluded from long-term follow-up: 4 patients had previous radiotherapy, 3 had previous ESWT, 4 had less than 3 sessions of ESWT, and 14 patients were lost of follow-up. Thus, we had sufficient data in 85 of the 110 patients.

### Baseline data (Table 2)

**Table 2:** Baseline data patients suffering from Peyronie’s disease treated by high-energy shockwave therapy using two different electromagnetic lithotripters

Parameter	Overall	Lithostar overhead module^a^ (group 1)	Lithoskop^a^ (group 2)
Patients treated	110	64	46
Patients evaluated	85	46	39
Median time of follow-up (months)	228 (6–288)	251 (288–226)	114 (6–226)
Plaque size (mm^2^)	89.9 (2–450)	90.4	88.8
Median penile deviation	35 (2–90)°	37°	35°
*N* < 30°	33	19	14
*N*: 30°–60°	42	23	19
*N* > 60°	10	4	6
Pain (VAS)			
In rest	2	2	2
During erection	6.7	6.5	7
During sexual intercourse	4.8	5.0	4.5
Erectile function			
IIEF-5 score	14 (11–16)	14	14
Severe and moderate impotence	59/85	32/46	27/39
Mild or no impotence	26/85	14/46	12/39
Peyronie’s disease score (PDQ-PS)	13 (11–17)	13	14

^a^Apart from the median time of follow-up no significant statistical differences between the two cohorts

**Fig. 1:**
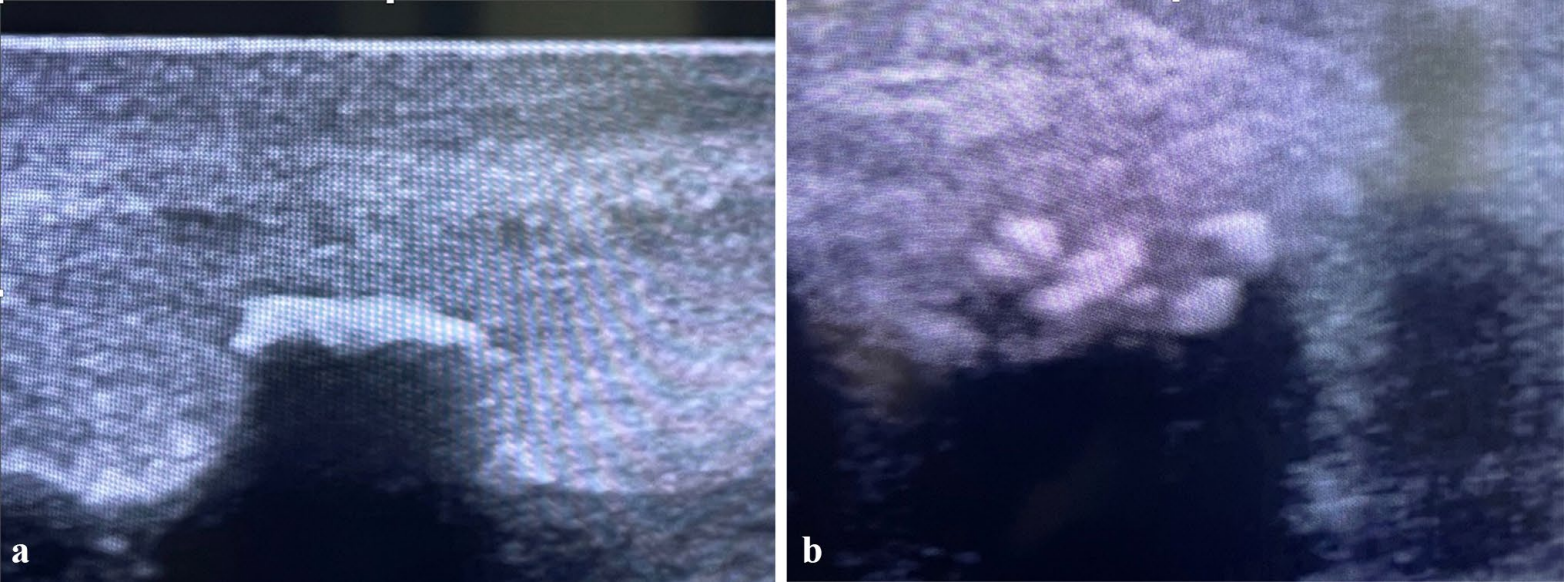
High-intensity extracorporeal shock wave therapy (ESWT) for management of Peyronie’s disease. Work-up of the patients. **a** Measuring of plaque size using 7.5 MHz-ultrasound probe. **b** Monitoring of treatment success using 7.5 MHz ultrasound probe: good fragmentation of plaque following three sessions at Siemens Lithoskop (40 MPa, 4000 SW, 0.4 mJ/mm^2^, 1 Hz)

The degree of curvature was determined using self-photography. We classified the patients according to their curvature in three categories (I: < 30°; II: 30–60°; III: > 60°). Plaque formation was determined by penile ultrasound (Fig. 1). Pain was evaluated using a visual-analog-scale (VAS). Erectile function was assessed using the International Index of Erectile Function-score (IIEF-5). Overall morbidity of the patients was evaluated by the use of a modified Peyronie’s Disease Questionnaire (PDQ) as proposed by Coyne et al. [22]. The instrument is comprised of three subscales: (1) Peyronie’s Psychological and Physical Symptoms subscale (six items); (2) Peyronie’s Symptom Bother subscale (six items); and (3) Penile Pain subscale (three items). In addition to our other methods of evaluation, we focused on Peyronie’s psychological and physical symptoms subscale (PDQ-PS).

### Lithotripters and ESWT regimen (Table 3)

**Table 3:** Long-term outcome of high-energy ESWT for Peyronie’s disease using two electromagnetic lithotripters

Parameter	Overall	Overhead^a^ module (group 1)	Lithoskop^a^ (group 2)
*N*	85	46	39
Median number of sessions	8 (3–14)	8 (3–14)	8 (4–12)
Median number of shocks	28,000 (9000–48,000)	30,000 (9000–48,000)	26,000 (12,000–40,000)
Acute complications			
Minor: skin hematoma (injection of lidocaine)	5 (5.8%)	3 (6.5%)	2 (5.1%)
Major	–	–	–
*Long-term outcome*			
General status			
Improved (PDQ-PS)	55 (65%)	31 (67%)	24 (61%)
Unchanged	16 (19%)	9 (20%)	7 (18%)
Worse	14 (16%)	6 (13%)	8 (21%)
Pain			
Improvement (VAS > 3)	20 (23.5%)	12 (26%)	8 (21%)
No pain	65 (76.5%)	34 (74%)	31 (79%)
Penile curvature			
Improved (> 5°)	43 (51%)	23 (50%)	20 (51%)
Unchanged	32 (37%)	18 (39%)	14 (36%)
Worse	10 (12%)	5 (11%)	5 (13%)
Erectile function			
Improved (IIEF-5 > 3)	40/59 (68%)	22/32 (69%)	18/27 (67%)
Unchanged	7/59 (12%)	4/32 (12%)	3/27 (11%)
Worse	12/59 (20%)	6/32 (19%)	6/27 (22%)
Penile surgery	9 (10.5%)	5 (10.8%)	4 (10.2%)
Plication	6	4	2
Collagene patch	2	–	2
Prothesis	1	1	–

*VAS* Visual analog scale, *IIEF-%* International Index of Erectile Function-score (Five items), *PDS* Peyronie’s disease score

^a^No significant statistical differences between the two cohorts

**Fig. 2:**
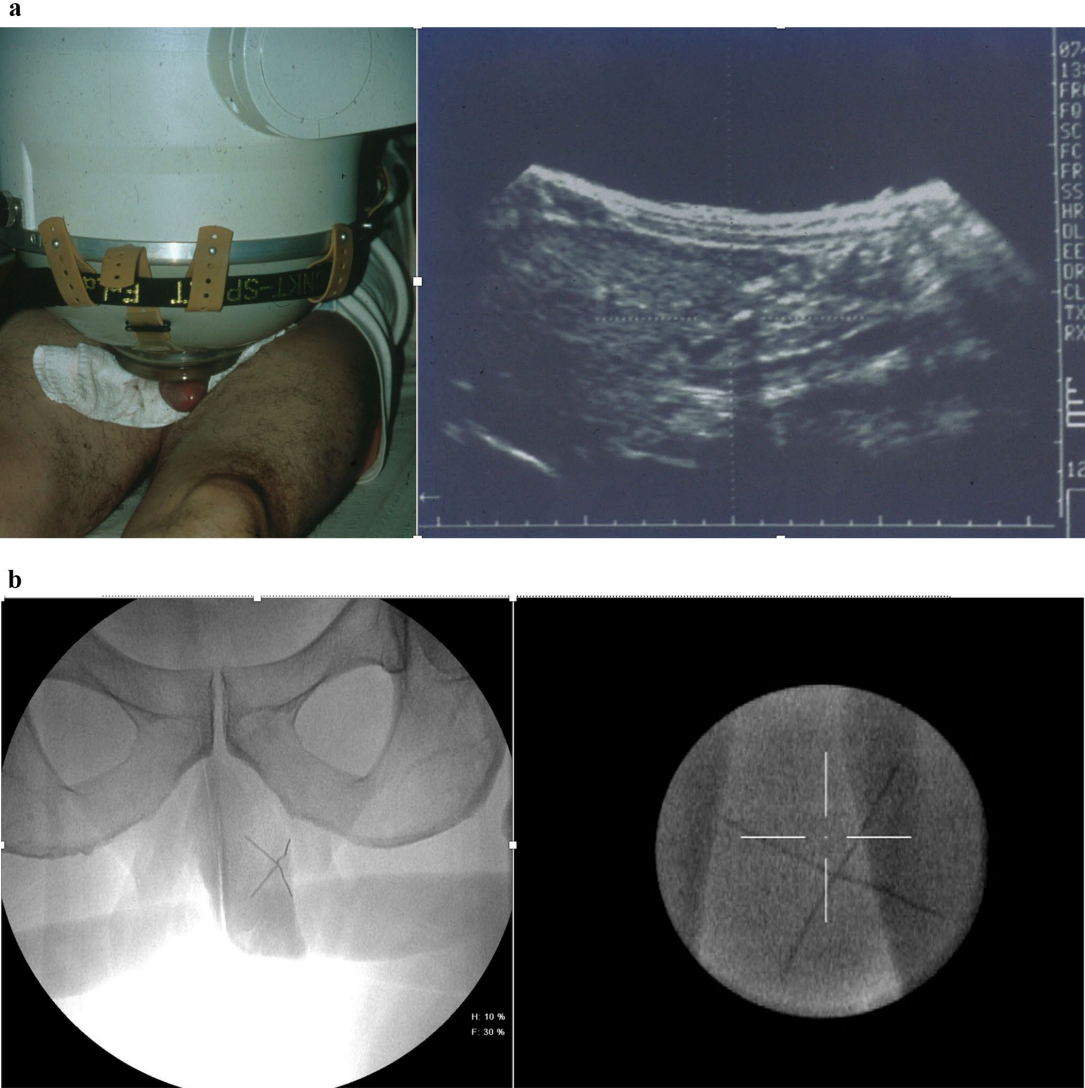
High-intensity extracorporeal shock wave therapy (ESWT) for management of Peyronie’s disease. Treatment technique. **a** Use of Siemens Lithostar Overhead Module with coupling device for extracorporeal shock wave lithotripsy in children. Ultrasonic localization of plaque. 21 kV, 3000–4000 impulses, level 6, 0.5 mJ/mm^2^, 1 Hz. **b** Use of Siemens Lithoskop with puncture of the plaque by two crossing needles (left) and in-line fluoroscopic localization (right)

Overhead Module of Lithostar Plus (Group 1; *n* = 64): ultrasound localization with the coupling device for children (Fig. 2a) applying three to 14 weekly sessions with 21 kV, 3000–4000 impulses, level 6, 0.5 mJ/mm^2^ at 1 Hz with a mean number of 30.000 (9.000–48.000). Siemens Lithoskop (Group 2; *n* = 46): Fluoroscopic localization of two crossing needles placed into the plaque (Fig. 2b) applying 40 MPa, 3000–4000 SW, 0.4 mJ/mm^2^, at1 Hz once a week with a mean number of 26.000 (12.000–40.000) impulses. All patients received local anesthesia via a penile block.

### Statistical analysis

Continuous variables are presented as the median and interquartile range (IQR) and were compared by Student’s independent *t* test or the Mann–Whitney *U* test based on their normal or not-normal distribution. Categorical variables were tested with the Chi-square test.

All statistical analyses were completed using Stata software, ver. 14 (StataCorp., College Station, TX, USA). For all statistical comparisons, a significance level of *p* < 0.05 was considered to show differences between the groups.

## Results

### Short-term results

Mean plaque size was 89.9 (2–450) mm^2^, median degree of penile deviation was 35 (2°–90°). Pain in rest was minimal (VAS = 2), however, all patients suffered from significant pain during erection (mean VAS = 6.7) and sexual intercourse (VAS = 4.8). 59 Patients had severe or moderate erectile dysfunction with a median IEEF-5-score of 14. Overall morbidity of the patients was reflected by a PDQ-PS-Score of 13 [11–17]. The baseline data of both groups were similar (Table 2).

We did not encounter any complications related to the application of shock waves. Under local anesthesia, the treatment was well tolerated. 5 Patients developed a superficial hematoma on the injection site.

### Long-term follow-up (Table 3)

Median follow-up was 228 (6–288) months and differed significantly between Group 1: 251 (228–226) months versus Group 2 114 (6–226) months. Pain reduction defined as a decrease of VAS-score by at least 3, was achieved in all 85 patients, of which 65 (76%) patients were completely free of pain. Improvement of penile curvature (ie. more than 5°) was achieved in 43 (51%) patients. The success depended on the baseline degree of deflection: Only 8 of 33 (24%) patients with a pre-treatment deflection angle < 30° experienced an improvement compared to 30 of 42 (71%) patients with a prior deflection angle of 30–60°. Deterioration of the penile curvature was observed in 10 patients (12%). The overall median deflection angle decreased from 35° to 20°.

59 patients reported problems with sexual intercourse, of those 40 (68%) reported improvement and 12 (20%) had deterioration of sexual function. The median IIEF-5-score increased from 14 to 21. Only 9 (10.5%) patients underwent surgical correction (6 × plication, 2 × graft interposition, 1 × penile prosthesis). Improvement of general status was reported by 55 (65%) of the patients, 14 (16%) patients reported deterioration. This was reflected by the increase of the PDQ-PS-score from 13 to 20. We did not encounter any significant differences between the electromagnetic lithotripters (Table 3).

### Comparison with baseline (Table 4)

**Table 4:** Comparison of outcome and baseline date of high-energy ESWT for Peyronie’s disease using two different electromagnetic lithotripters

Parameter	Baseline	Outcome	*p* value
Median penile deviation	35 (2–90)°	20 (2–90)°	< 0.05
Pain (VAS)			
In rest	2	0.25	< 0,05
During erection	6.7	0.5	< 0.05
During sexual intercourse	4.8	0.5	< 0.05
IIEF-5 score	14 (11–16)	21 (16–25)	< 0.05
General status (PDQ-PS-score)	13 (11–17)	8 (6–12)	< 0.05

*VAS* visual analog scale, *IIEF-%* International Index of Erectile Function-Score (Five items), *PDS* Peyronie’s disease score

Comparing the overall baseline results with the follow-up data, we observed a significant improvement in all categories (Table 4). Accordingly, no significant differences between the two Lithotripters could be detected.

## Discussion

Peyronie’s disease represents the first urological indication for extracorporeal shock wave therapy [5, 6]. Based on experimental studies of Seemann and Haupt [23, 24] on the effect of shock waves on wound and fracture healing, ESWT was introduced for orthopedic indications, such as the treatment of pseudarthrosis, tendinopathy, and tendinosis calcaria [25]. At this time, we started with the treatment of chronic phase Peyronie’s disease using the Overhead Module of the Siemens Lithostar Plus [5]. The initial idea was to break the plaques similar to extracorporeal shock wave lithotripsy.

Very soon, manufacturers presented devices specifically designed for orthopedic indications. These devices were much easier to use and applied significantly lower amounts of shock wave energy. Thus, the principle of low-intensity shock wave therapy (Li-ESWT) with an energy flux density of < 0.3 mJ/mm^2^ has been developed. The main advantage of these devices represents the fact, that ESWT can be applied without any anesthesia under palpatory control.

In 2009, Vincenzo Mirone’s group presented the first randomized controlled study using the Storz Duolith [7]. They used an energy flux density of 0.25 mJ/mm^2^ at 4 Hz applying 4000 impulses weekly with a total of 4 sessions. After 12 weeks, pain, erectile function and quality of life ameliorated significantly in patients receiving ESWT. In 2010, Chitale et al. [12] published the results of a second RCT comparing low-energy ESWT with a sham-treated control group. The type of device used was not even stated in the article. 3000 SWs were delivered at level 25 (38 MPa) with 4 Hz for the SWT group in 6 weekly sessions and the same number of SW in the sham group but at level 0. Only 36 patients were included. The authors did not find any differences between both groups. However, these results should be taken with caution due to the poor design of the study. Recently, Sokolakis et al. [20] presented the long-term results of their randomized controlled trial using the Piezoson 100. All patients were treated with an energy flux density of 0.29 mJ/mm^2^ at 3 Hz applying 3000 shocks weekly for 6 weeks. They could confirm their 3 months results with a significant reduction of pain, which persisted at 3 years [9, 20].

Unfortunately, no further randomized controlled trials have been presented. Thus, recent EAU guidelines recommend Li-ESWT only for the treatment of pain associated with PD [2]. However, we should still consider several non-randomized studies, which were able to demonstrate more efficacy of ESWT in Peyronie’s disease [26, 27]. Our results confirm these studies with high efficacy on pain and significant improvement of the penile curvature in 51% of the patients resulting in improvement of erectile dysfunction in 68%. Only 9 (10.5%) patients underwent penile surgery compared to 23% in the study of Siranganam et al. [19] applying low-dose ESWT using the Storz Minilith. Also, Strebel et al. [14] reported a better efficacy on penile curvature when using the Siemens multiline lithotripter compared to the Dornier EPOS Ultra device and the Storz Minilith SL 1. We observed, that the degree of deflection had an impact on the result: we saw only 25% improvement in patients with an angle of less than 30°), whereas 95% of patients with an angle of 30°–60° responded well. Our study has the longest follow-up of all published series. It demonstrates the stability of the response. The three patients who were pre-treated with ESWT were all non-responders. This is in accordance with the other long-term studies [19–21].

Initially, we found the Overhead Module of the Siemens Lithostar Plus ideal, because we could localize the plaque by ultrasound and monitor the course of treatment [5]. However, the device was taken off the market. Nevertheless, we could successfully continue with the use of the Siemens Lithoskop under fluoroscopic guidance. A significant advantage of the new devices, such as the Storz Duolith SD represents the fact, that there is no need for anesthesia and the shock wave application can be performed under palpatory control.

The main question concerning the design of future studies should be based on the hypothesis of the working mechanism of extracorporeal shock waves. In 2002, Mirone et al. [28] presented a very interesting study performing a cone biopsy of the cavernous tissue. 380 patients with chronic PD were treated either with ESWT 3 times a week for 20 min followed by a complete cycle of 12 intralesional verapamil 10 mg injections (group A) or had only the verapamil injection cycle serving as the control group (group B; *N* = 92). In those 260 patients with a successful outcome of ESWT, histological evaluation of the specimens revealed a reduction in packing and clumping of the collagen fibers. This resembles recent experimental data from Tom Lue’s group using the rat model of birth trauma [29]. Also, recent findings on the possible activation of stem cells might be included in the healing process [30].

Moreover, it is obvious, that PD does not represent a stable but phase-wise continuing disease and even within the same phase PD may present with different clinical features [26]. These huge interindividual differences in PD manifestations make it almost impossible to use one fixed ESWT protocol with fixed energies and sessions to fit all the PD patients. Because of this variability among the different PD-patients we all are still in an experimental phase to adjust the energy, number of shots and sessions, and the devices and applicators preferred [26]. Finally, the recently published study of Spirito et al. [21] showing promising and stable long-term results applying Li-ESWT for acute phase Peyronie’s disease may open a new window for extracorporeal shock wave lithotripsy: It should be tested in a randomized trial against medical treatment (i.e. Potaba or Vitamine E) rather than waiting for the chronic phase of the disease.

This study represents a retrospective non-randomized study and the number of applied sessions varied between 3 and 10 sessions. Moreover, our results will not be reproducible, because both lithotripters are no longer produced by the manufacturer Siemens. Nevertheless, they provide important information for the design of future trials focusing on the efficacy of extracorporeal shock wave lithotripsy on Peyronie’s disease. Definitively the use of high-energy-ESWT has to be included.

## Conclusions

High-energy shock wave therapy delivered by two standard electromagnetic lithotripters is safe and efficient providing stable long-term results. In cases with significant plaque formation, the concept of high-energy ESWT should be considered in future studies.

## Data Availability

All data presented would be available for further studies.
